# Moran KS, Gold CL, eds. Forensic Archaeology: Multidisciplinary Perspectives 1st edition. Springer; 2019. 333 pages. ISBN 978-3-030-03289-0 (hardcover), ISBN 978-3-030-03291-3 (e-Book)

**DOI:** 10.3325/cmj.2019.60.297

**Published:** 2019-06

**Authors:** Vedrana Petrovečki

**Affiliations:** Institute of Forensic Medicine and Criminalistics, University of Zagreb School of Medicine, Zagreb, Croatia *vedrana.petrovecki@mef.hr*

**The Field of Medicine:** Forensic medicine

**Format:** Hardcover book, e-Book

**Audience:** This book is of interest particularly to forensic pathologists, as well as to other professionals involved in the investigational process, such as forensic anthropologists and police investigators.

**Purpose:** Forensic archeology is the newest discipline in the family of forensic disciplines. A couple of events influenced the recent development of forensic archeology in the UK and Europe: the war on the territory of former Yugoslavia, especially in Bosnia and Herzegovina, with a large number of mass graves victims requiring identification, and numerous homicide cases in mid- to late-1990s in the UK, whose efficient solution demanded the involvement of academic archeologists. As opposed to Europe, forensic archeology is still less recognized in the US, and formal education in forensic archeology exists only as a part of academic courses on forensic anthropology.

Some improvements have been made at the beginning of 21st century, when forensic archeology was first recognized among archeologists, leading to the establishment of a small session dedicated to forensic archeology at the annual meeting of the Society for American Archeology’s in 2004. At the same meeting in 2013, there were 23 separate communications presented in three sessions, and some of the presented material is included in this book. Further recognition of forensic archaeology requires active dissemination in relevant forensic science journals and meetings.

The book aims to familiarize the audience with this new forensic discipline and to extend the knowledge of the experts interested in this particular field. The book advocates the recognition of forensic archeology as a standard forensic discipline, which would allow full implementation of archeological techniques, and the involvement of trained archeologists in the field investigation.

**Content:** The book consists of four parts. The first part, entitled “Theoretical Framework,” in its five chapters aims to determine the position of forensic archeology within the forensic sciences. It emphasizes the importance of the recognition of forensic archeology as a separate discipline from forensic anthropology, with equal importance in forensic investigation, complementary to forensic anthropology. This chapter also emphasizes important differences between classical and forensic archaeology. Forensic archeology developed through the application of anthropological methods and techniques in crime scene investigation applicable in both domestic and international settings and the cases of human remains repatriations or the cases of mass disasters. So, forensic archeology is not just an academic, but also a stabile applied discipline. The important topic discussed is the need for education of forensic archeologists. The prevailing attitude is that the UK model should be used, in which forensic archeologists should receive their degree in archeology, but should be familiar with police procedures, sampling protocols, and the corresponding administrative work. A fundamental insight is essential in the whole process, from fieldwork to court, in addition to excavation skills, basic knowledge about human anatomy and osteology, and forensic science (forensic medicine, forensic toxicology, serology, DNA, fingerprinting, etc). The presented case study clearly demonstrates the application of these skills and knowledge during crime scene investigation.

The second part, “Forensic Archeological Context,” focuses on archeological terminology that applies to forensic archeology in the field- and court-related purposes. It emphasizes the necessity of developing precise terms and definitions in order to improve forensic archeologists' presentation and contribution to court hearings as experts. In this part, several chapters are also dedicated to the collaboration between forensic archeologists and law enforcement officers, presented through case descriptions from forensic practice. This collaboration has proved useful in establishing the predicting models to locate missing persons. Especially interesting was the involvement of volunteers from Forensic Archeology Recovery, an organization of students and staff from Brown University, in old cases of missing persons, a volunteering that is hard to imagine in our society. The presented cases also clearly demonstrate the position and importance of archeological techniques and methods in the investigation of human remains at landfills and forensic fire scenes.

The third part entitled “Multi-disciplinary Techniques and Methods,” deals with the role of palynology and entomology in forensic archeological investigation, and emphasizes the role of chemists in this type of investigations. Through several examples, it explains and illustrates the role of 3D laser scanning technology in improving the archeological damage investigation, and additionally addresses the role of statistical methods and procedures in the field.

The fourth part includes case studies, which authors use to provide a detailed insight into the importance and specific roles of forensic archeology in the investigation process.

**Highlights:** Careful reading of this book will provide the reader with the clear definition of forensic archeology and its distinction from forensic anthropology. Authors state that “physical anthropology is much deeper than creating a biological profile, and archeology is more than just digging” and that forensic archeology needs to be established as “the field as separate but equal to forensic anthropology”.

